# Dynamic Surgical Restoration of Mid and Lower Facial Paralysis: A Single-Greek-Centre Experience

**DOI:** 10.7759/cureus.52387

**Published:** 2024-01-16

**Authors:** Foteini Neamonitou, Maria Kotrotsiou, Spyros Stavrianos

**Affiliations:** 1 Plastic Surgery, General Anticancer Oncological Hospital of Athens Agios Savvas, Athens, GRC; 2 Plastic and Reconstructive Surgery, Evangelismos General Hospital, Athens, GRC; 3 Plastic and Reconstructive Surgery, Saint Savvas Hospital, Athens, GRC

**Keywords:** facial nerve, facial palsy, facial paralysis, myoplasty, reconstruction

## Abstract

Background

Facial palsy detrimentally impacts an individual’s quality of life due to its effects on function and appearance. There are several reconstructive surgical techniques available that aim to restore facial symmetry. Techniques such as direct neurorrhaphy, nerve grafts, dual reanimation, and reinnervation have the potential to enable varying motor functions, including the re-establishment of a dynamic smile. This study aimed to assess the outcomes of facial palsy reconstructive surgeries undertaken at a tertiary care centre for facial nerve reconstruction in Athens.

Methods

This study consisted of a comprehensive case series showcasing the outcomes of facial palsy reconstructive surgeries on 29 patients at our Tertiary General Oncological Anti-Cancer Hospital of Athens ’Agios Savvas’. The surgical procedures from October 2004 to December 2023 included reinnervation, nerve grafting, free muscle transfer, and myoplasties following our recommended algorithm. We categorized the patients into two groups: Group A and Group B based on the timing of the reconstruction: delayed or immediate. The House-Brackmann grading scale evaluated the degree and improvement of facial paralysis.

Results

In Group A, two of the seven patients exhibited activation of the mimetic musculature immediately postsurgery, while the remaining five experienced enhanced facial nerve function in the subsequent months. Adverse outcomes were temporalis dysfunction in one case and tongue atrophy in another. Conversely, in Group B, 21 of 22 patients demonstrated facial activation immediately postsurgery. Only one patient from this group did not show any facial nerve function postoperatively. Two of the 22 patients in Group B encountered complications: one with trismus and another with temporalis dysfunction. All patients were observed for a minimum of 12 months postsurgery.

Conclusion

With the exception of one patient, all participants showed improved postoperative results, which were satisfactory when weighed against the observed morbidity rate. While our case analysis did not reveal any clear indication of one particular technique being superior, the selection of methods should be based on several factors, and this algorithm could serve as a useful aid in that regard. A comprehensive and standardized clinical assessment of facial palsy, both before and after surgery, is crucial to establish a consensus and plan individualized therapy.

## Introduction

Facial palsy, a multifaceted condition, often profoundly affects an individual’s physical and emotional well-being. This disorder may lead to functional challenges, including difficulties in swallowing, articulation, and lip insufficiency, paired with aesthetic issues like facial asymmetry. Plastic surgery provides a range of reconstructive procedures tailored to the individual’s specific needs, restoring movement, sensation, and facial symmetry. It is crucial to note that nerve healing progresses gradually; sensory nerves usually recover more rapidly than motor nerves. On average, sensation might take up to three years to return, while motor recovery generally ceases after 12 to 18 months. Axonal growth is estimated at 1 mm per day, or approximately 1 to 3 cm per month [1].

The surgical strategy for facial reconstruction often demands a comprehensive approach. Key factors like the injury’s severity, the extent of facial paralysis (partial or complete), availability of a distal nerve stump, functional facial muscles, and the time elapsed since the injury shape the clinical assessment. Following this, electromyography (EMG) and conductivity studies are used to craft a treatment strategy. This strategy addresses functional issues, re-establishes facial symmetry, promotes spontaneous movement and addresses post-paralysis facial synkinesis.

Preoperative assessment necessitates a clinical evaluation of the smile, often aided by the House-Brackmann (HB) grading scale [2]. The HB facial nerve grading system is an established clinical tool for the standardized assessment of facial nerve function, primarily in conditions such as Bell's palsy, trauma, or postoperative scenarios. The HB scale classifies facial nerve dysfunction into six grades, ranging from I, indicative of normal function, to VI, which corresponds to complete paralysis. Grade I represents normal facial movement in all areas; Grade II denotes slight dysfunction with minimal synkinesis; Grade III is characterized by obvious but not disfiguring weakness; Grade IV includes obvious disfiguring weakness with severe synkinesis or contracture; Grade V signifies severe dysfunction with barely perceptible motion; and Grade VI reflects an absence of detectable facial movement.

Furthermore, a speech assessment and cranial nerve examination are indispensable for determining donor nerve viability. Examining facial and temporal arterial pulses helps identify recipient vessels. Electrophysiological probes can help anticipate patients with partial acute palsy outcomes within the first 18 months [3]. Options for reconstruction encompass techniques like direct neurorrhaphy, nonvascularized nerve graft bridging, myoplasties, and free muscle transfer. This study aimed to assess the outcomes of facial palsy reconstructive surgeries undertaken at our department, the sole Tertiary Reconstructive Centre for facial nerve reconstruction in Athens.

Cross-face nerve graft (CFNG) has emerged as a leading reconstructive choice for patients with irreversible moderate to severe facial nerve palsy, especially when the injury’s denervation is less than six months old. Whether administered as a single or dual-stage procedure, CFNG holds promise for restoring spontaneous smiles. For denervation durations of six months to one year, the babysitter technique is recommended, which combines CFNG with partial hypoglossal transfer. This technique aids in preserving facial musculature and mitigates the complications of cranial nerve XII cross-over [4]. However, CFNG’s effectiveness remains under scrutiny. While some clinicians prefer the “babysitter” technique for injuries that have lasted over six months before intervention [5], others argue that single-stage CFNG can produce outcomes similar to the two-stage method but with fewer procedures [6].

Regarding procedures involving the hypoglossal nerve, a common strategy involves entirely sectioning and attaching the hypoglossal nerve to a facial nerve branch. Although this approach can reinstate facial tone and voluntary movement, it may introduce complications like difficulties in speech, chewing, and swallowing. For those experiencing synkinesis and facial contraction, partial neurotomy of the hypoglossal nerve could be more suitable [7]. However, alternative methods, such as interpositional grafts or splitting the hypoglossal nerve, have their own constraints and risks [8,9].

The masseteric motor nerve transposition technique is gaining attention due to its robust reinnervation capabilities and minimal donor site side effects. Executing this method demands detailed dissection of the masseteric nerve while preserving its proximal branches to prevent masseter muscle paralysis or deterioration [10-14]. When compared to CFNG, masseteric nerve myoplasty shows enhanced results in facial symmetry, recovery time, and overall success in single-stage procedures [15-17].

The lengthening temporalis myoplasty (LTM) technique is renowned for its simple surgical execution, especially when the distal nerve stump is missing. LTM builds upon Gillies’ seminal work from 1934 [18] with further advancements since then [19-21].

The dual reanimation techniques are distinctive for merging two restoration strategies to regain control of denervated facial muscles, aiming for dynamic smile restoration. The domain of dual innervation is also growing, with techniques that employ double-innervated free gracilis muscle transfer taking precedence [22,23]. Nonetheless, the application and success of dual innervation techniques demand further extensive research to draw firm conclusions [24,25].

## Materials and methods

**Figure 1:**
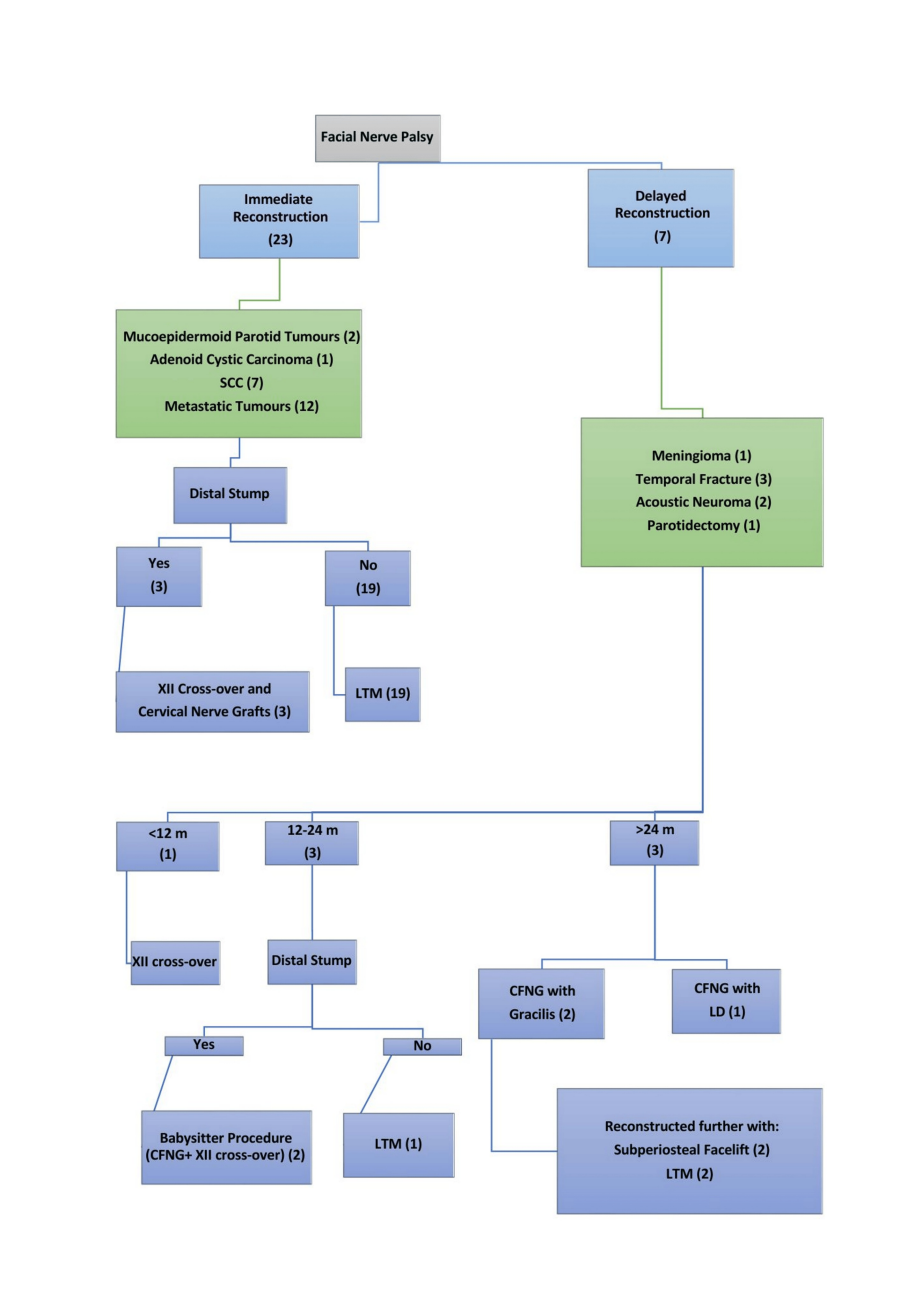
Algorithm of facial reconstruction LTM, lengthening temporalis myoplasty; LD, latissimus dorsi flap; CFNG, cross-face nerve graft.

We retrospectively analyzed a systematically maintained patient database from the General Oncological Anti-Cancer Hospital of Athens’ Agios Savvas’ from October 2004 to December 2023. All patients consented to the publication of their data after receiving local approval from our institution’s audit department (Approval No. 6976/18-03-2021). Based on the timing of the reconstruction, patients were classified into two categories: Group A for delayed and Group B for immediate reconstruction. Surgical decisions hinged on factors such as the duration post-injury, the state of the distal nerve stump, and the degree of muscle atrophy, as visualized in a summation algorithm (Figure 1).

The study assessed two distinct patient groups. Group A consisted of seven patients who underwent delayed reconstruction. Group B comprised 22 patients who underwent immediate reconstruction following our team’s oncological procedures. We assessed each patient’s level of impairment both pretreatment and six months post-treatment using the HB scale. We used the visual analogue scale (VAS) to assess the satisfaction of the patient in Group A. An interdisciplinary team spearheaded by the senior author and plastic surgeon consultant, comprising plastic surgery residents, specialized nurses, physiotherapists, and speech and language therapists, played a pivotal role in this research.

## Results

Analysis of Group A

We analyzed seven patients, aged 20 to 72 years, with an average age of 39.4 years, who underwent reconstructive surgery for facial nerve injuries. The causes of these injuries were traumatic temporal fractures in three patients, meningioma surgery in one patient, acoustic neuroma surgeries in two patients, and parotidectomy in one patient.

Using the HB scale, all patients were categorized as Grade VI (indicating complete facial nerve deficiency). The duration between the onset of paralysis and the reconstructive surgery varied from nine months to seven years, with an average of 37.7 months. All patients underwent a preoperative needle EMG, which indicated moderate to severe facial nerve injuries and muscle fibrillations. The EMG results demonstrated moderate to complete denervation within the facial nerve boundary, evidenced by absent motor unit action potential recruitment and nerve trunk inexcitability.

Reconstructive surgery decisions depended on the time since the injury. Consequently, patients were divided into three time-based categories: less than 12 months, 12 to 24 months, and more than 24 months. Among the three patients with temporal fractures, CFNG reconstruction was performed for all. Two of these received additional free muscle transfers (one with a latissimus dorsi muscle and another with a gracilis muscle) due to muscle atrophy. The remaining patient, possessing a viable distal stump, underwent a cranial nerve XII cross-over. The patient who had meningioma surgery received a CFNG combined with a gracilis muscle transfer. Of the two patients with acoustic neuroma, one was treated with the babysitter technique (incorporating both CFNG and partial XII transfer), while the other underwent XII cross-over. Lastly, the patient who had parotidectomy received a temporalis myoplasty reconstruction. Two patients who initially received CFNG and gracilis muscle transfer due to persistent resting asymmetry and unsatisfactory dynamic smile excursion later underwent further reconstruction incorporating a subperiosteal facelift and temporalis myoplasty.

All patients were monitored for 12 months to seven years. Of the seven, two demonstrated noticeable activation of the mimetic musculature immediately after surgery, while the remaining five displayed enhanced facial nerve functionality after several months. The average onset of the initial contraction occurred within three months postsurgery (ranging from 0 to 15 months). Typically, the first observable mimetic musculature contractions were at the nasolabial fold.

**Figure 2:**
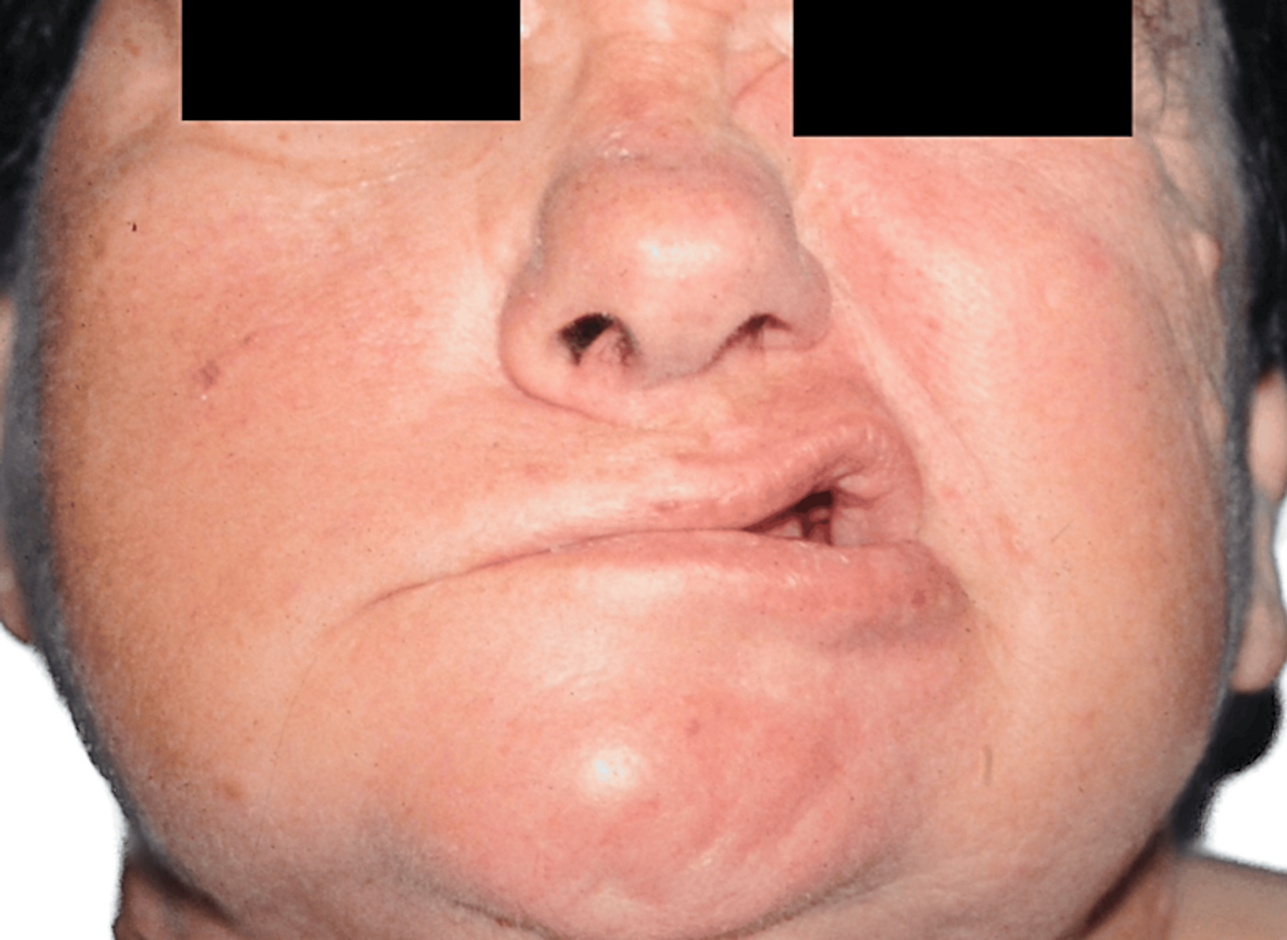
Patient A, 52 y/o female with a history of meningioma before reconstruction 52 y/o female with a history of meningioma before reconstruction y/o, years old

**Figure 3:**
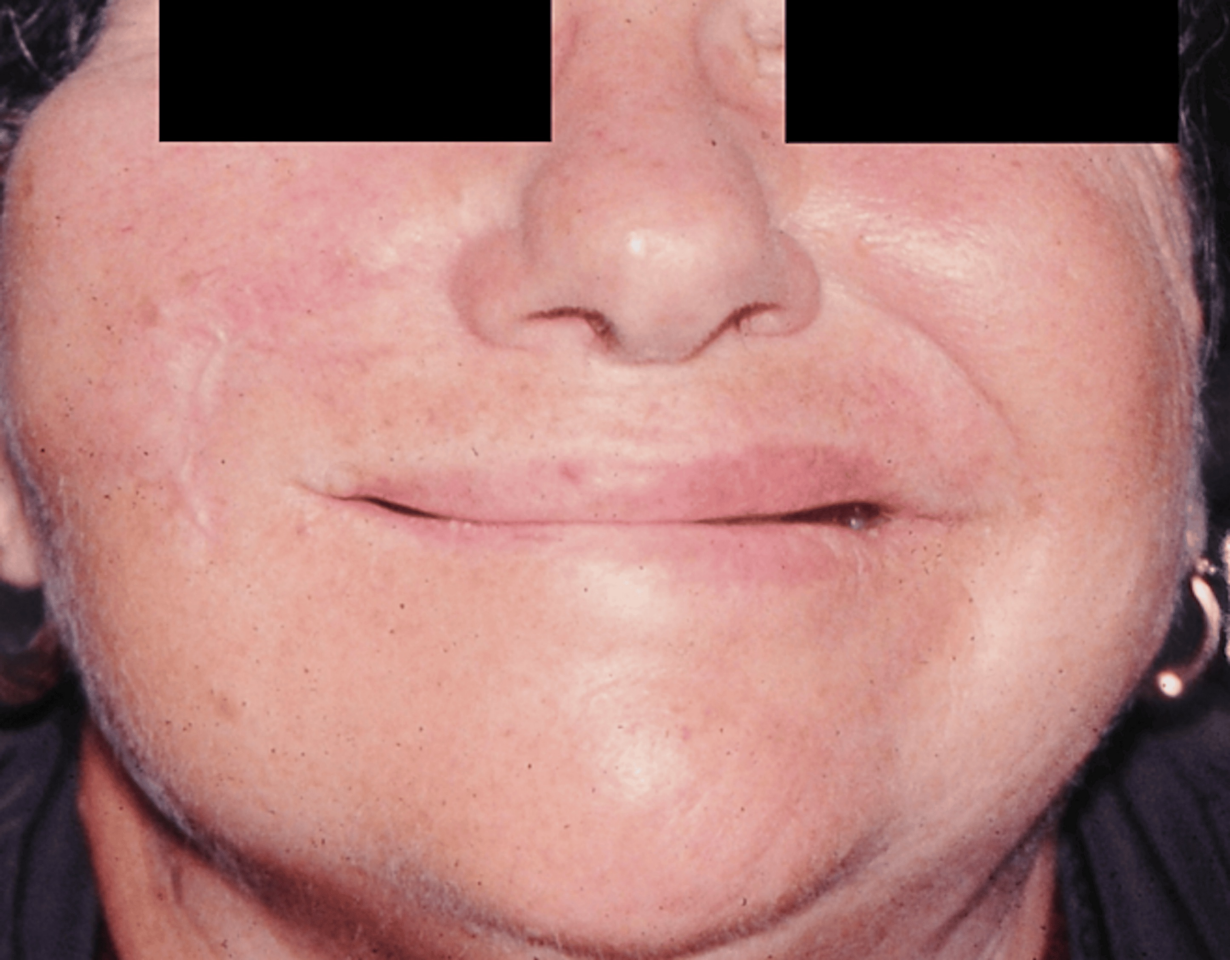
Patient A, postoperative view after one year of reconstructive surgery The patient had received CFNG and gracilis muscle transfer and later LTM and subperiosteal facelift. CFNG, cross-face nerve graft; LTM, lengthening temporalis myoplasty

**Figure 4:**
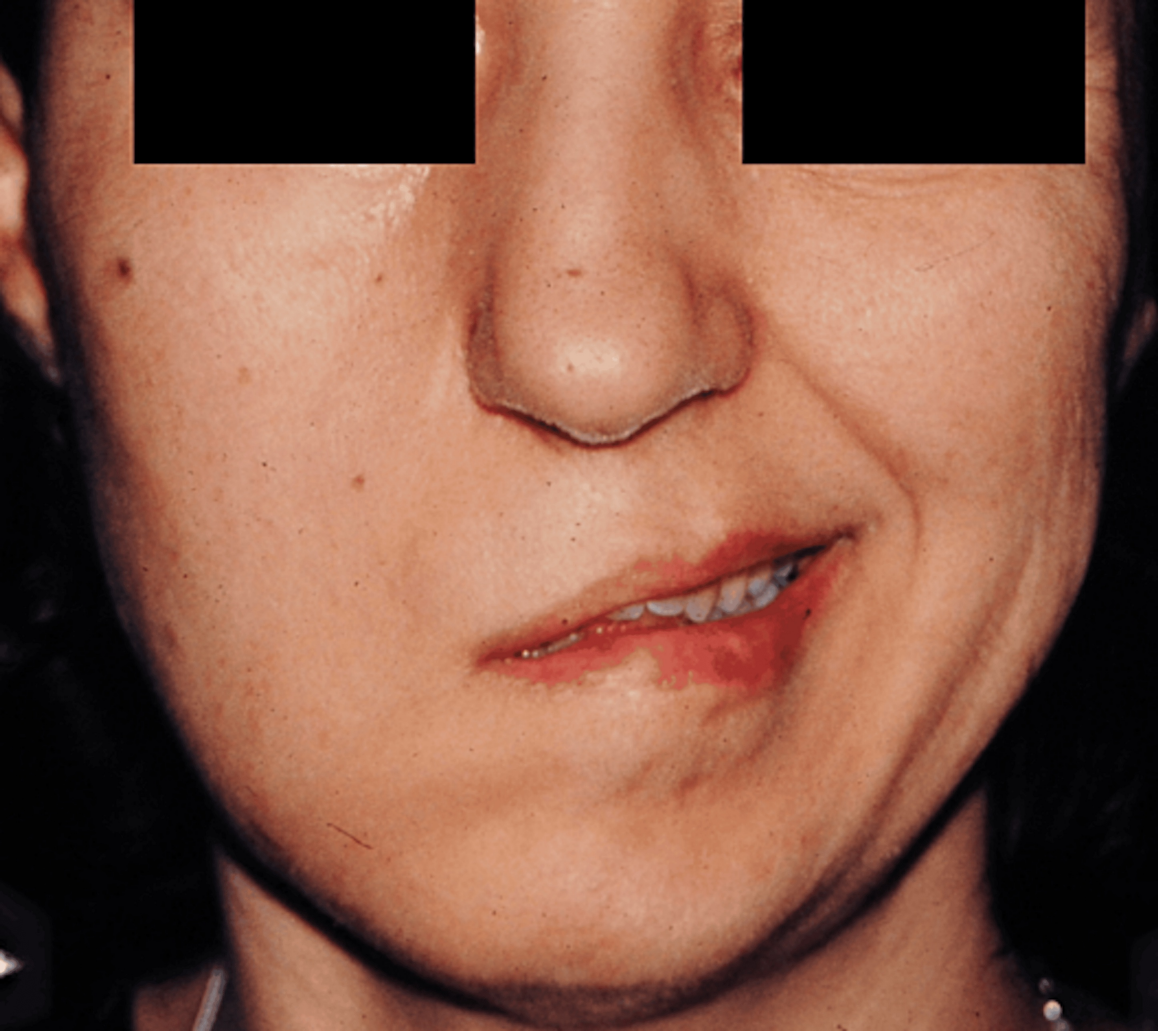
Patient B, 43 y/o female with history of traumatic temporal fracture and petrosal bone injury, preoperative view y/o, years old

**Figure 5:**
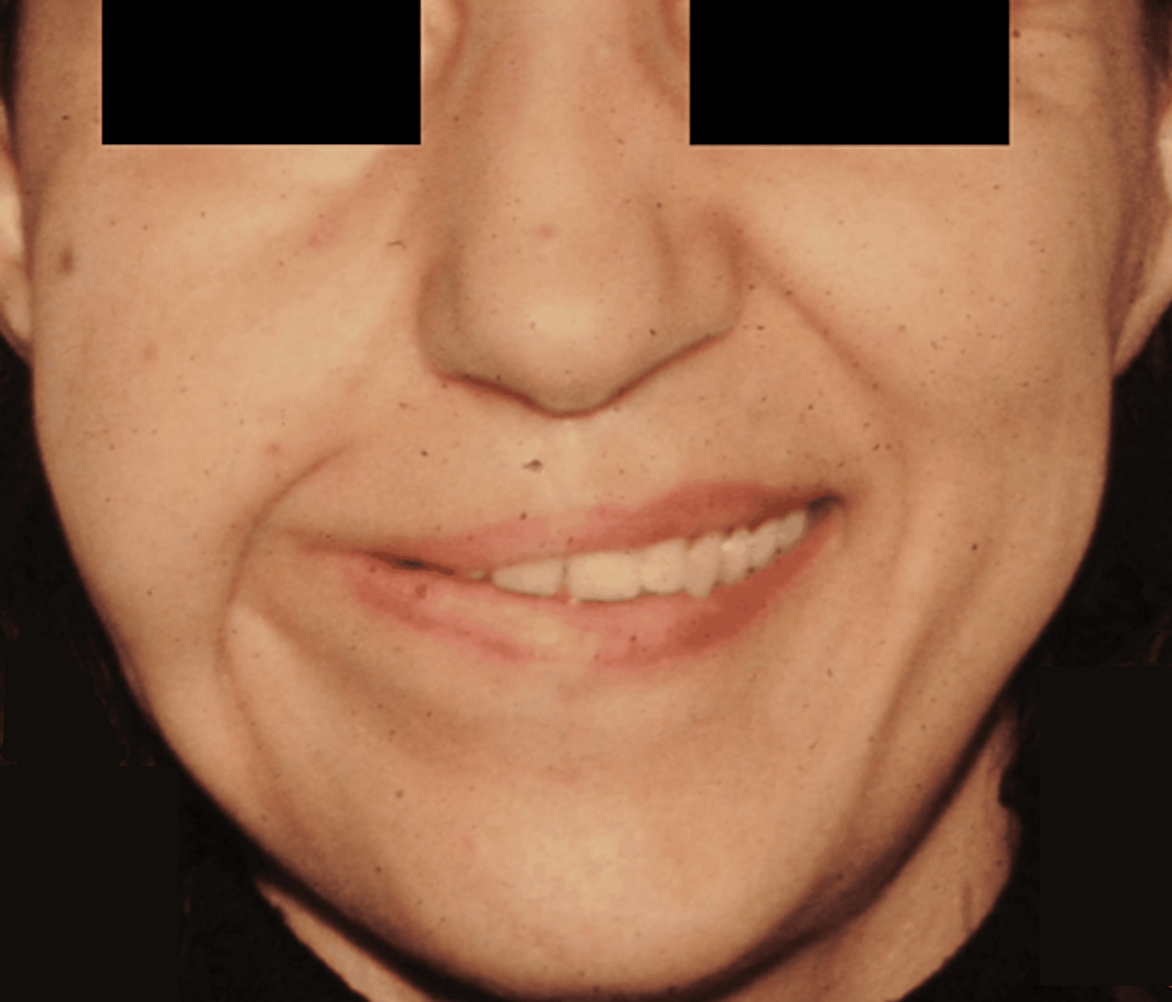
Patient B, postoperative view after 15 months of reconstructive surgery The patient had received CFNG and gracilis muscle transfer and later LTM and subperiosteal facelift. CFNG, cross-face nerve graft; LTM, lengthening temporalis myoplasty

**Figure 6:**
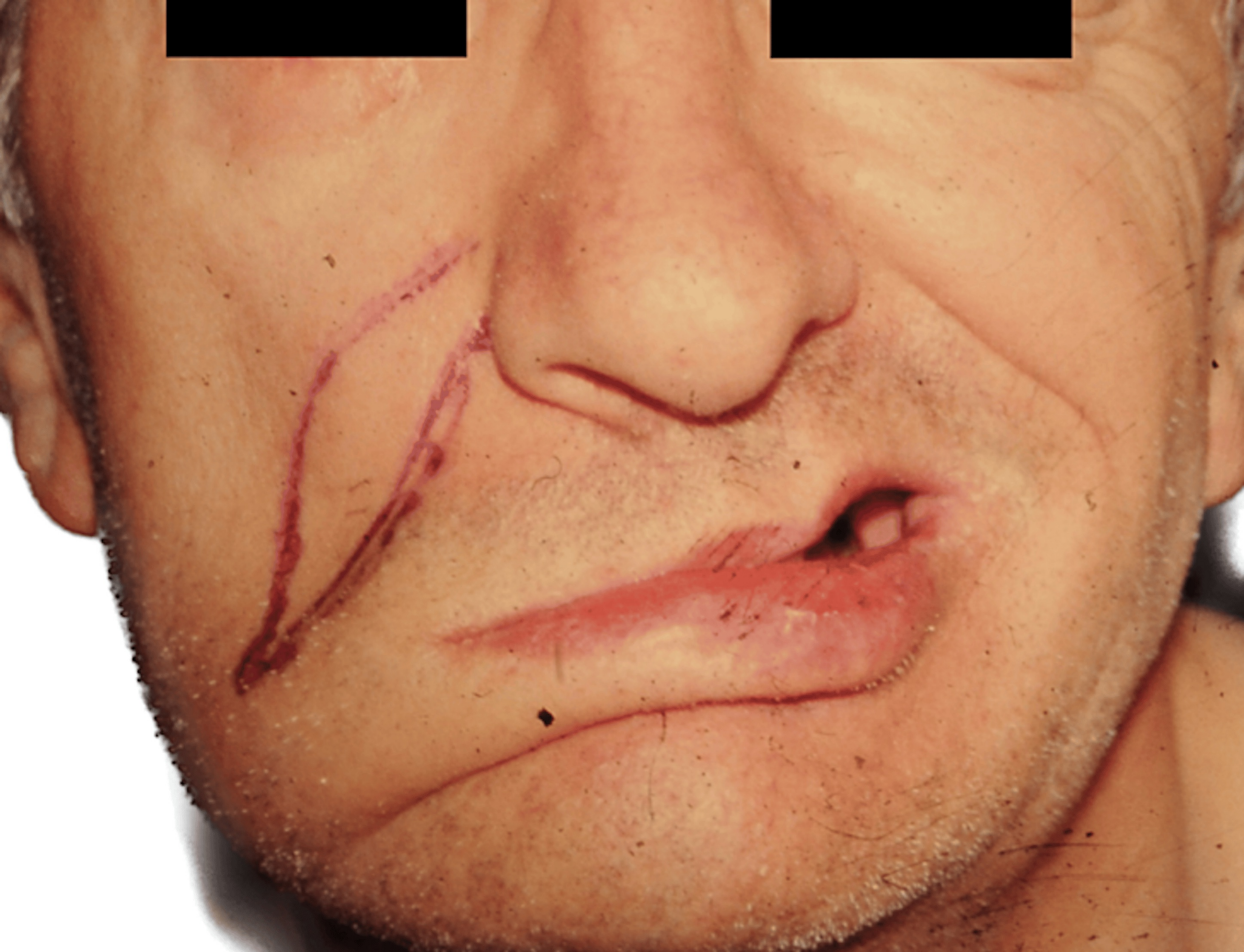
Patient C, 65 y/o male with traumatic temporal fracture, preoperative view y/o, years old

**Figure 7:**
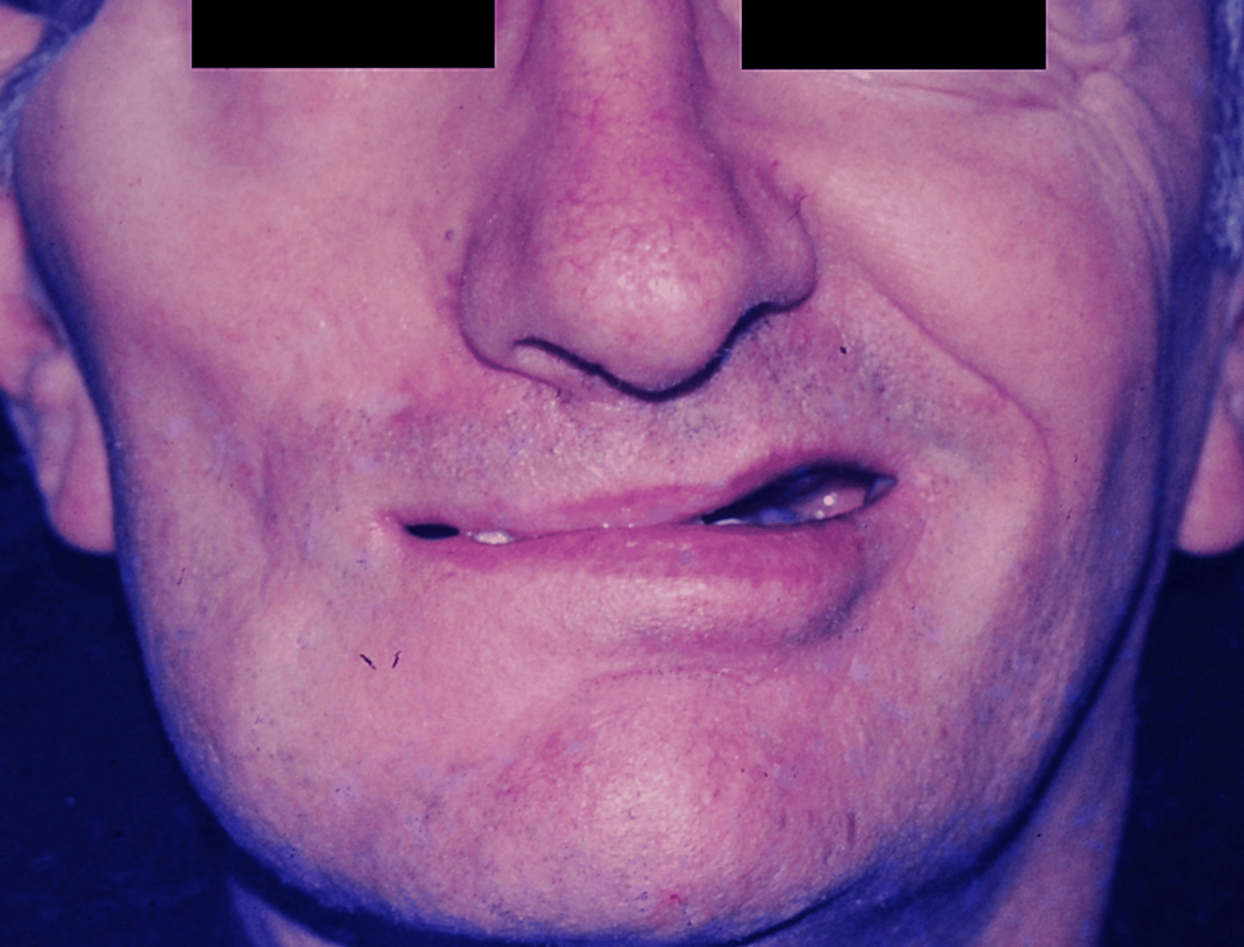
Patient C, postoperative view after one year of reconstructive surgery The patient had received CFNG and latissimus dorsi muscle transfer CFNG, cross-face nerve graft

Eighteen months after the initial visible contraction, the modified HB grading scale classified one patient as Grade III, five as Grade IV and one as Grade V (Table 1). Two patients experienced complications: one had temporalis dysfunction, and the other had partial tongue atrophy following temporalis myoplasty and XII cross-over, respectively. The patient with partial tongue atrophy received speech therapy. As part of the patient assessment, a visual analogue scale was used to evaluate their overall level of satisfaction. The majority of patients reported satisfactory outcomes (7.4/10), with only one patient scoring a 5 on the scale (Figures 2-7).

**Table 1:** Group A case series HB, House-Brackmann, CFNG, cross-face nerve graft; LTM, lengthening temporalis myoplasty; VAS, visual analog scale score; PreOp, preoperative; PostOp, postoperative. ^a^Time between the onset of paralysis and reconstructive surgery

Patient No.	Age in years, Sex	Timing^a^ (years, months)	Etiology	Type of Reconstruction	HB Grade		VAS	
					PreOp	PostOp	PreOp	PostOp
1	43, F	6	Traumatic Temporal Fracture	CFNG & Gracilis Muscle Transfer / LTM & Subperiosteal Facelift	6	4	1	8
2	52, F	7	Meningioma	CFNG & Gracilis Muscle Transfer / LTM & Subperiosteal Facelift	6	4	1	8
3	65, M	4	Traumatic Temporal Fracture	CFNG & Latissimus Dorsi Muscle Transfer	6	4	1	8
4	20, M	1, 9	Traumatic Temporal Fracture	Babysitter procedure	6	4	1	8
5	40, F	1	Acoustic Neuroma	Babysitter procedure	6	3	1	7
6	52, M	0, 9	Acoustic Neuroma	XII cross-over	6	4	1	8
7	72, M	1	Parotidectomy	LTM	6	5	1	5

Analysis of Group B

**Figure 8:**
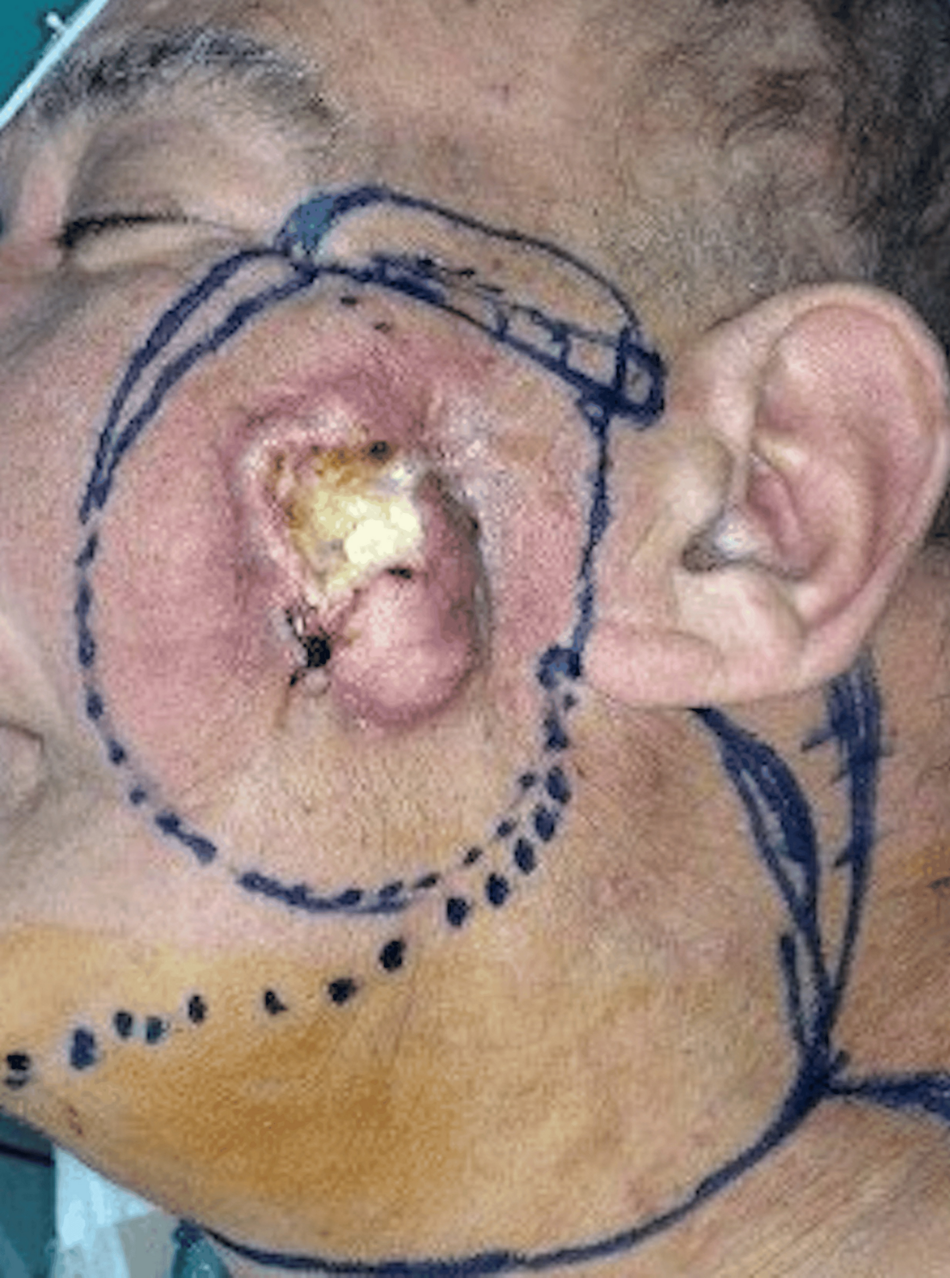
Patient D, 67 y/o male with cSCC, preoperative view cSCC, cutaneous squamous cell carcinoma

**Figure 9:**
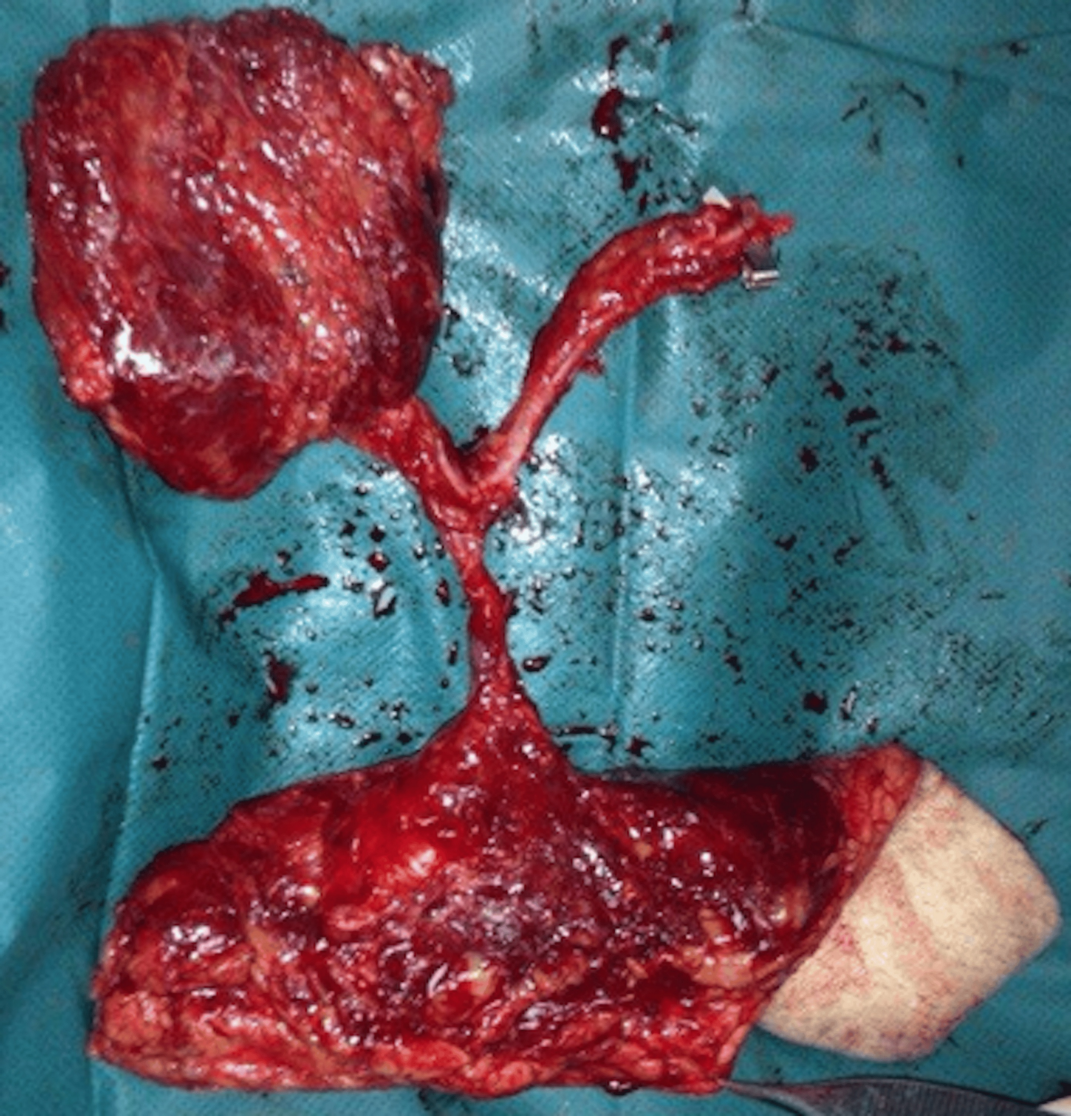
ALT+ VL, intraoperative view ALT, anterolateral thigh flap; VL, vastus lateralis

**Figure 10:**
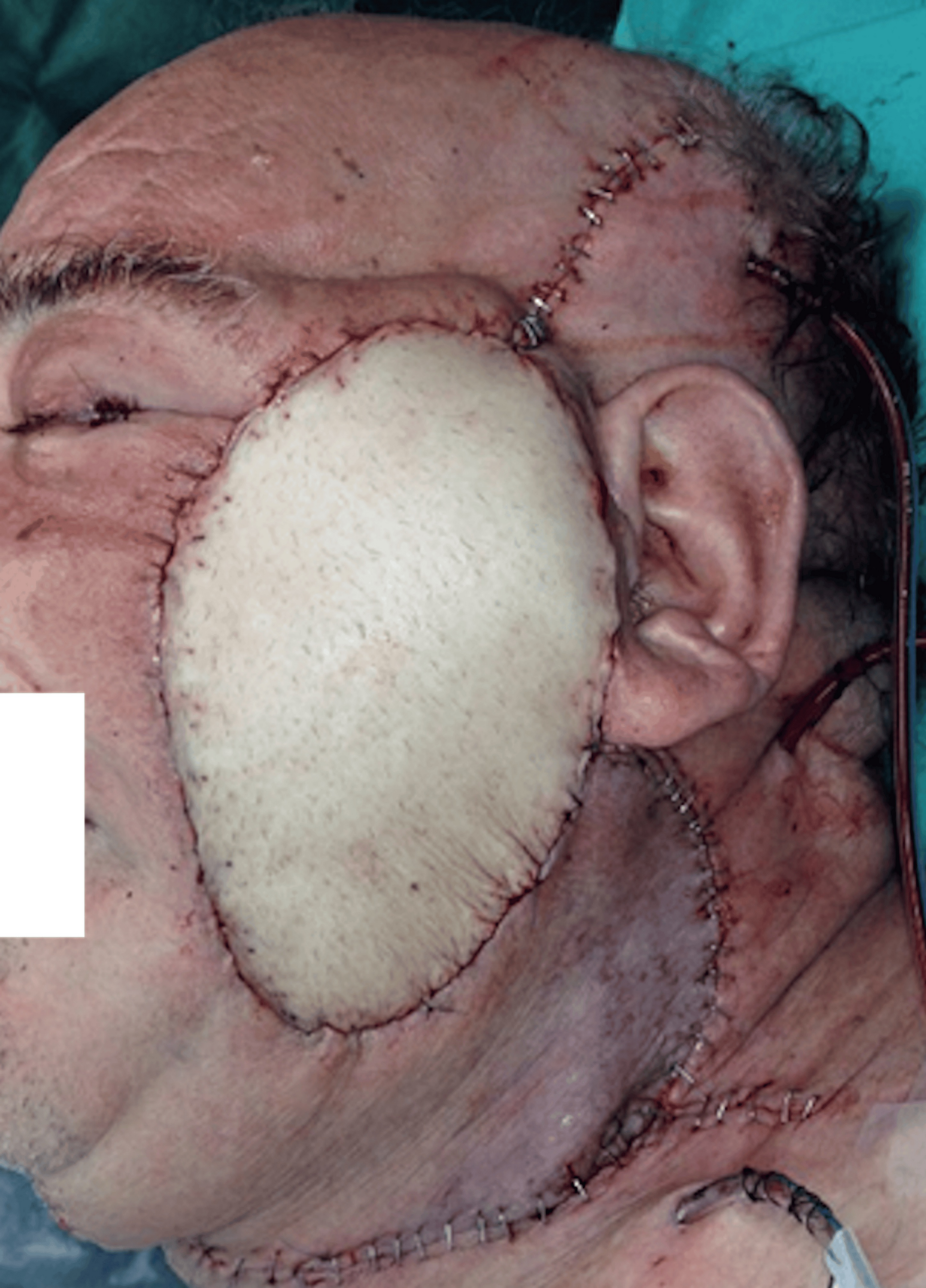
Patient D, immediate postoperative view

Group B consisted of 22 patients aged 42 to 72 years (mean age 62.4). Group B patients underwent immediate reconstruction after removal of benign or invasive tumours from the head and neck, which required the resection of the facial nerve or its branches. If these patients had not undergone reconstruction, they would have experienced complete paralysis of the facial nerve, as it had been completely severed (Figures 8-10).

This group included six patients with facial cutaneous squamous cell carcinomas (cSCCs), two with mucoepidermoid tumours of the parotid, one with an acoustic neuroma, one with adenoid cystic carcinoma, six with metastatic parotid cancers, and six with tumours from the skull-base, orbital region, and maxilla.

The six cSCC patients were reconstructed using LTM and three of those patients required additional reconstruction with free tissue transfer. These cases required free tissue transfer for covering large defects. Therefore, anterolateral thigh flap (ALT) with vastus was used (Figure 8-10). The two mucoepidermoid and the single adenoid cystic carcinoma cases underwent XII cross-over and cervical nerve graft procedures. All metastatic parotid tumour patients, along with the individual with acoustic neuroma and those with skull base cancers (13 in total), received temporalis myoplasty (Table 2). The patients with skull-based tumours received LTM with free tissue transfer to reconstruct the large defects, close the skull base cavities, and prevent CSF leak. These cases were reconstructed with ALT with or without vastus lateralis (VL), free fibula flap (FFF), and free radial forearm flap (FRFF).

In the immediate postsurgery phase, 17 patients showed facial activation, four began contracting within a month postsurgery, and one did not exhibit any facial nerve function. Employing the modified HB grading scale, we classified four patients as Grade III, sixteen as Grade IV, two as Grade V, and one patient (with adenoid cystic cancer) as Grade VI one year after their first visible contraction. All patients were followed up for at least 12 months. During this period, two patients faced complications: trismus and temporalis dysfunction. Both patients were diagnosed with metastatic parotid tumours and are scheduled to receive gracilis free flap reconstruction along with the patient with complete paralysis.

**Table 2:** Group B case series cSCC, cutaneous squamous cell carcinoma; HB, House-Brackmann, PreOp, preoperative; PostOp, postoperative; LTM, lengthening temporalis myoplasty; ALT, anterolateral thigh flap; VL,vastus lateralis; FRFF, free radial forearm flap; FFF, fibula free flap.

Patient No.	Age in years, Sex	Etiology	Type of Reconstruction	HB Grade	
				PreOp	PostOp
1	62, F	cSCC	LTM	2	4
2	70, M	cSCC	LTM	3	4
3	69, M	cSCC	LTM	3	4
4	72, M	cSCC	LTM & ALT + VL	2	4
5	67, F	cSCC	LTM & ALT+ VL	3	4
6	55, M	cSCC	LTM & ALT+VL	3	5
7	67, F	Mucoepidermoid tumors	XII cross-over & cervical nerve grafts	2	5
8	60, F	Mucoepidermoid tumors	XII cross-over & cervical nerve grafts	2	3
9	42, F	Acoustic neuroma	LTM	3	4
10	53, F	Adenoid cystic cancer	XII cross-over & cervical nerve grafts	3	6
11	62, M	Metastatic parotid tumors	LTM	3	3
12	61, M	Metastatic parotid tumors	LTM	3	4
13	64, F	Metastatic parotid tumors	LTM	3	3
14	57, M	Metastatic parotid tumors	LTM	2	4
15	55, M	Metastatic parotid tumors	LTM	3	4
16	55, F	Metastatic parotid tumors	LTM	2	4
17	67, M	Orbital tumor	LTM & FRFF	3	4
18	72, F	Orbital tumor	LTM & FRFF	2	4
19	70, M	Maxillary tumor	LTM & ALT	3	4
20	70, F	Maxillary tumor	LTM & ALT	3	4
21	59, F	Maxillary tumor	LTM & FFF	2	4
22	63, M	Maxillary tumor	LTM & ALT+VL	2	3

## Discussion

Facial reanimation techniques have steadily advanced with a primary goal of restoring functional and aesthetic outcomes for patients affected by facial paralysis. A key indicator of success is restoring a spontaneous and symmetrical smile, which is crucial for addressing the emotional and psychological repercussions of paralysis. Various techniques have emerged for dual reanimation strategies, including nerve grafts, CFNG, the masseteric motor nerve transposition method, lengthening temporalis myoplasty, cranial nerve XII-to-VII coaptation and free tissue transfer. Each of these techniques comes with merits and challenges.

Our literature review revealed that patients who receive treatment within 18 months of their injury are more likely to have favourable outcomes than those who begin treatment at a later stage [26]. Our patients showed quicker recovery and good outcomes with spontaneous smile with immediate or early reconstruction within one year, as supported by a systematic review of patients with traumatic facial nerve palsies [27].

In contrast to these findings, our results demonstrate that modern neurotization techniques can significantly enhance recovery from long-standing facial paralysis. In most longstanding cases where a successful mandibular smile has been achieved, the spontaneity of the smile was problematic. Treatment success depends on a personalized approach. Our results suggest multifactorial outcomes, with many promising treatment methods [28].

To provide the best possible care for patients with facial palsy, standardized clinical evaluations must be performed before and after surgical interventions. The HB scale is widely used in the assessment of facial nerve paralysis. Other rating scales, such as the Sunnybrook or Sydney systems, and even computer-aided systems, have been developed to aid in the surgical evaluation of facial palsy. Despite their availability, these tools are not commonly applied in standard clinical follow-ups. Volk et al. recommended a diagnostic protocol to steer these critical assessments [29]. The rigour of these evaluations is crucial; it advances the field, fosters agreement among clinicians and researchers, and enhances the calibre of forthcoming studies.

Facial reanimation techniques present both advantages and disadvantages that are distinct from one another. Among them, lengthening temporalis myoplasty (LTM) is commonly used due to its simplicity and short duration compared to microneurorhaphies. Additionally, it causes less damage to the donor site and eliminates the need for repositioning the patient during surgery, making it a desirable option. Moreover, it can aid in covering tissue defects after the removal of cancers. According to Al Khabori et al., a three-step rehabilitation process after LTM can achieve spontaneous smiles, making it a reproducible technique with excellent results [30]. Microneurorrhaphies can result in a more natural-looking smile, but they may not always be feasible due to technical complexities, time that has passed since the injury, and muscle viability. Free neurotized muscle transfer is a complicated yet effective procedure used to treat long-lasting paralysis. This procedure demands good technical skills, an additional donor site, and a longer operative time. Each technique has its own specific indications; however, achieving a spontaneous smile with a single procedure remains debatable. Therefore, utilizing dual or triple reanimation procedures in selective cases could potentially lead to even better outcomes and higher success and satisfaction rates.

Our study had a few important limitations. First, the sample size was relatively small, potentially reducing the power of our analyses and the generalizability of our findings to a broader population. Additionally, the retrospective nature of our research design might have introduced selection bias and limited our ability to identify potential confounding factors. We also relied on patients’ self-reporting for certain data points, which might be subject to recall bias. Conducting more thorough individual assessments could aid in evaluating patient satisfaction. Furthermore, the lack of a control group makes it challenging to ascertain the efficacy of the procedures relative to standard treatments or interventions. Lastly, the short follow-up period in some cases may not truly capture long-term outcomes and complications associated with the surgical procedures.

## Conclusions

Facial paralysis profoundly affects a patient’s emotional and physical health, often leading to disfigurement. After analyzing our case series, we did not find any evidence to suggest that any particular techniques were superior overall. Apart from one patient, all experienced satisfactory postoperative improvements with a low complication rate. Fortunately, recent advancements in facial reconstruction offer the possibility of individualised treatment for the best possible outcome with natural facial nerve rehabilitation.
