# Correction: Dynamic Surgical Restoration of Mid and Lower Facial Paralysis: A Single-Greek-Centre Experience 

**DOI:** 10.7759/cureus.c212

**Published:** 2025-02-18

**Authors:** Foteini Neamonitou, Maria Kotrotsiou, Spyros Stavrianos

**Affiliations:** 1 Plastic Surgery, General Anticancer Oncological Hospital of Athens Agios Savvas, Athens, GRC; 2 Plastic and Reconstructive Surgery, Evangelismos General Hospital, Athens, GRC; 3 Plastic and Reconstructive Surgery, Saint Savvas Hospital, Athens, GRC

This article has been corrected where the word “proximal” was used multiple times incorrectly. It has been changed to reflect that the nerve stump is “distal” in the Introduction, results of group A, and Material and Methods. Figure 1 has also been updated to reflect the distinction as “distal”.

